# Functional Reorganization of the Central Auditory System in Children with Single-Sided Deafness: A Protocol Using fNIRS

**DOI:** 10.3390/brainsci12040423

**Published:** 2022-03-22

**Authors:** Marie-Noëlle Calmels, Yohan Gallois, Mathieu Marx, Olivier Deguine, Soumia Taoui, Emma Arnaud, Kuzma Strelnikov, Pascal Barone

**Affiliations:** 1 Service d′Oto-Rhino-Laryngologie, d′Oto-Neurologie et d′ORL Pédiatrique, Centre Hospitalier Universitaire de Toulouse, CEDEX 9, 31059 Toulouse, France; gallois.y@chu-toulouse.fr (Y.G.); marx.m@chu-toulouse.fr (M.M.); deguine.o@chu-toulouse.fr (O.D.); taoui.s@chu-toulouse.fr (S.T.); kuzma.strelnikov@cnrs.fr (K.S.); 2 Centre de Recherche cerveau et Cognition, Université de Toulouse, Université Paul Sabatier, 31052 Toulouse, France; arnaudemma1@gmail.com (E.A.); pascal.barone@cnrs.fr (P.B.); 3 UMR 5549, Faculté de Médecine de Purpan, Centre National de la Recherche Scientifique, 31055 Toulouse, France

**Keywords:** fNIRS, single-sided deafness, unilateral hearing loss, pediatrics, functional neuroimaging, plasticity, cortical reorganization

## Abstract

In children, single-sided deafness (SSD) affects the development of linguistic and social skills and can impede educational progress. These difficulties may relate to cortical changes that occur following SSD, such as reduced inter-hemispheric functional asymmetry and maladaptive brain plasticity. To investigate these neuronal changes and their evolution in children, a non-invasive technique is required that is little affected by motion artifacts. Here, we present a research protocol that uses functional near-infrared spectroscopy (fNIRS) to evaluate the reorganization of cortical auditory asymmetry in children with SSD; it also examines how the cortical changes relate to auditory and language skills. The protocol is designed for children whose SSD has not been treated, because hearing restoration can alter both brain reorganization and behavioral performance. We propose a single-center, cross-sectional study that includes 30 children with SSD (congenital or acquired moderate-to-profound deafness) and 30 children with normal hearing (NH), all aged 5–16 years. The children undergo fNIRS during monaural and binaural stimulation, and the pattern of cortical activity is analyzed using measures of the peak amplitude and area under the curve for both oxy- and deoxyhemoglobin. These cortical measures can be compared between the two groups of children, and analyses can be run to determine whether they relate to binaural hearing (speech-in-noise and sound localization), speech perception and production, and quality of life (QoL). The results could be of relevance for developing individualized rehabilitation programs for SSD, which could reduce patients’ difficulties and prevent long-term neurofunctional and clinical consequences.

## 1. Introduction

Binaural hearing involves various mechanisms that analyze and integrate auditory information from the two ears. Interaural disparities are particularly important, as they facilitate the comprehension of speech in noise and sound localization. Impaired binaural hearing occurs when there is single-sided deafness (SSD) or bilateral asymmetric hearing loss. This has significant socio-behavioral consequences and can impede educational progress in the case of children.

Functional magnetic resonance imaging (fMRI) studies in adults with SSD have shown a reduction in the functional asymmetry of the auditory cortices. This has also been shown for children with bilateral profound deafness who have been treated with a unilateral cochlear implant; this reduced asymmetry persists following cochlear implantation of the remaining side if the delay is longer than 1.5 years.

To the best of our knowledge, there is little data concerning the consequences of untreated SSD at the cortical level in the case of children.

Here, we hypothesize that SSD in children modifies the auditory cortical activation profile, and that this relates to (i) binaural hearing skills, (ii) language development, and (iii) quality of life (QoL).

### 1.1. Background

Unilateral hearing loss (UHL) is a common hearing disorder that can be either congenital or acquired, and it affects 0.5–5% of children of all ages [1,2,3]. UHL is two to three times more common than bilateral hearing loss and its incidence is increasing [4]. Before the systematic screening of newborn hearing, UHL was rarely diagnosed before the age of five, and then only during routine screening at schools [5]. Newborn hearing screening has revolutionized the diagnosis of UHL and has shown that 0.05–4% of neonates are affected [6,7,8].

#### 1.1.1. UHL Consequences and Care

In the 1970s, UHL was not considered to be a disability and its management was limited to identifying the underlying causes and advising children’s parents [9,10]. Research carried out since the 1980s has caused this to change. This work showed that UHL in children can affect auditory skills, language, educational levels, and behavior [11]. In the case of auditory skills, impairments were shown for auditory discrimination, sound localization, and speech-in-noise comprehension [12,13,14,15]. In the case of language skills, several studies showed negative effects on the verbal IQ [16,17,18,19] and on tests assessing morphology, syntax, and expressive vocabulary [20]. The studies that assessed educational levels revealed a range of difficulties that affected 34–78% of the children [16,21]. Behavioral difficulties were also reported by some studies, with teachers perceiving problems in 20–59% of children with UHL [8,12]. This wide range of problems can lead to a decrease in the quality of life of children with UHL [21,22,23].

Recommendations for treating UHL in children were published by the American Academy of Audiology in 2013 [24]. Despite these recommendations and the accumulating evidence for UHL-related difficulties, many children are not offered an adapted treatment plan [25]; treatment is often only considered for children with delayed language acquisition. However, several treatment options are available, as for bilateral deafness. Moderate to severe hearing loss can benefit from a conventional hearing aid, although this depends on the condition of the external auditory canal and pinna. Conductive hearing loss can be treated with a bone conduction hearing aid [26,27], which uses a device attached to a headband for children younger than five or an osseointegrated device such as a BAHA (Bone Anchored Hearing Aid) for older children. In the case of severe deafness, the auditory information that reaches the deaf ear can be transferred to the healthy ear through a wireless system (CROS System: Contralateral Routing of Signal) or bone (BAHA) conduction, leading to “pseudo” binaural hearing. Cochlear implantation (CI) is also used for UHL rehabilitation, but this is currently restricted to adults with profound deafness and disabling tinnitus that is unresponsive to conventional treatment [28,29,30]. However, in 2019, the US Food and Drug Administration approved cochlear implantation for children with single-sided deafness (SSD) [31].

There is now a large body of evidence demonstrating the benefits of treating SSD in adults, regardless of the rehabilitation strategy. These benefits include improved speech intelligibility in noise (signal-to-noise ratio, SNR), sound localization, and quality of life (QoL) [28,32,33]. Arndt et al. [34] assessed the auditory skills of a group of patients with SSD, first with pseudo binaural hearing (CROS and then BAHA on a headband), and then 6 months following CI. It was found that sound localization and speech comprehension in noise were much better following the CI. More recently, a prospective, multi-center study [30] demonstrated that QoL improves following CI in adults with unilateral or asymmetric profound deafness and severe tinnitus.

Treatment of UHL in children has also shown satisfactory levels of recovery, as in adults. In the case of conductive hearing loss, there have been encouraging results for BAHAs, with improved speech perception in noise [26]. For moderate to profound UHL, there is evidence that CROS or bone conduction hearing devices improve the pure tone average and speech recognition in quiet or noise [35]. A recent meta-analysis by Benchetrit et al. [36] showed that CI for children with profound SSD was associated with clinically meaningful improvements in auditory skills (sound localization and speech perception in quiet and noise) and patient-reported outcomes (for speech recognition, spatial hearing, and hearing quality, as assessed using a questionnaire). Similarly, a meta-analysis by Nicolas et al. [35] showed improved quality of life following treatment for UHL using various devices in children. However, the failure rate of UHL treatment is high, regardless of the rehabilitation method adopted, with failure rates ranging from 21 to 75% [22,23,37]. Individual outcomes are very variable, both in terms of binaural performance and subjective quality of life. The paucity of robust clinical data specific to pediatric patients is problematic when advising families concerning the clinical treatment options and their potential advantages. In this context, it is important to understand the cortical reorganization that occurs following SSD, particularly in children, and the implications that this has for the mechanisms involved in brain plasticity. This is important, as these processes could underlie some of the variability seen in the rehabilitation outcomes. This information could be used to guide clinical decision making concerning the rehabilitation of children with SSD.

#### 1.1.2. Cortical Reorganization Following Unilateral Deafness

In adults

The auditory system is strongly lateralized along the different levels of processing. In normally-hearing subjects, monaural stimulation induces the hemodynamic activation of the contralateral auditory cortex, a pattern that results from uneven projections from the cochlea to the contralateral hemisphere [38,39]. This lateralization of auditory information processing is present at all levels of the auditory pathway [40] and is referred to as contralateral dominance. It has been suggested that this lateralization may relate to processing auditory information from the contralateral sound field rather than the contralateral ear per se [41,42,43].

Studies have been run using animal models to investigate cortical reorganization following early SSD [44,45]. These have shown that auditory-induced neuronal activity shifts towards the hemisphere ipsilateral to the normally-hearing ear, with both increased excitability/activity and a reduced latency. This cortical reorganization towards the functional ear has been described as “aural preference syndrome” and is thought to be characteristic of congenital unilateral deafness [44].

In humans, the cortical reorganization that results from prolonged hearing loss can be investigated using different functional brain imaging methods, such as electroencephalography (EEG), magnetoencephalography (MEG), near-infrared spectroscopy (NIRS), magnetic resonance imaging (MRI), and positron emission tomography (PET). In adults with SSD, brain imaging studies have shown decreased interhemispheric asymmetry following stimulation of the preserved ear. Specifically, monaural stimulation induces a more pronounced activation of the contralateral auditory cortex in normally-hearing subjects, while there is more bilateral activation for subjects with SSD; this has been shown using fMRI [46,47,48] and EEG/MEG [49,50]. The relevance of the side of the deafness, right or left, was studied by Hanss et al. [49]. They found that the normal lateralization pattern is more strongly affected by left-sided deafness. In SSD patients after therapeutic cochlear nerve resection, the shift from contralateral dominance to more bilateral patterns can take several months [50,51], thus suggesting that the shift in lateralization is a dynamic mechanism. It is possible that this process may relate to how well individual patients adapt to having only monaural auditory input. For instance, there is evidence that some adult patients with UHL develop adaptive strategies that enable them to perform at near-normal levels on both sound localization and speech-in-noise comprehension tasks [52,53]; this is likely to result from patients learning to use head shadow effects as well as monaural spectral cues [54,55]. One study showed that the auditory adaption relates to the extent of the shift in cortical lateralization, with better spatial hearing scores being related to a smaller ipsilateral shift [56]. Another study found that the restoration of binaural inputs to patients with SSD through CI restored the contralateral pattern of auditory activation as well as improving sound localization and speech-in-noise performance [57].

During development

Few studies have investigated cortical reorganization in children with SSD or asymmetric hearing loss. An anatomical study using MRI track-density imaging found that children with SSD had altered connectivity within the auditory pathway, revealed by decreased fractional anisotropy values. This may reflect a maturational delay [58] due to the lack of peripheral input.

Animal studies have been carried out to investigate the impact of congenital SSD on the lateralization of auditory cortical activity [44,59]. These studies, carried out on cats, have confirmed that there is a stronger representation of the hearing ear in the cortex ipsilateral to that ear. Similar findings have been reported for children with SSD [60] and also for children with bilateral deafness following sequential bilateral CI [61], thus strengthening the evidence for an aural preference syndrome. A study by Sharma et al. [62] showed a deficit in auditory cortical maturation in a child who received sequential bilateral CI, which was restricted to the cortex contralateral to the second implanted ear; of note, this second implant was received after the age of seven, while the first was received by the age of 3.5. In another study, Gordon et al. [63] presented EEG evidence for cortical reorganization in children with bilateral profound deafness treated with CI. When the second cochlear implant was received after a delay of 1.5 years or more, there was an abnormal ipsilateral aural preference that persisted over time, even after prolonged experience with the bilateral implants. This abnormal pattern related to a speech perception deficit for the second implanted ear [64]. In contrast, when the second cochlear implant was received after a delay of less than 1.5 years, the cortical responses showed a similar contralateral pattern for both ears [63]. The same research group [60,65] also showed an abnormal aural preference in children with early SSD, which was partially reversed by restoring binaural inputs through CI. The lateralization patterns and their modification after CI were highly variable and may have related to the duration of deafness and experience with the implants.

#### 1.1.3. fNIRS Brain Imaging in Children with UHL

It can be challenging to measure brain activity in children. fMRI is not usually suitable because of the length of time participants are required to remain still, which is typically around 50 min (57). EEG studies require a large number of trials, which can be long and tedious for children; in addition, this technique has limited spatial resolution, for adults as well as for children, because EEG relies on algorithms of source reconstruction that lack accuracy. Invasive methods and those using radioactive markers, such as PET, are also unsuitable for pediatric populations.

In the present protocol, we use fNIRS to measure functional brain activity in children. fNIRS assesses cortical activity levels using spectroscopy; specifically, it detects an increase in blood flow to active cortical areas by assessing the concentrations of oxyhemoglobin and deoxyhemoglobin [66]. This is performed using two different near infra-red wavelength photonic beams, each specific to the type of hemoglobin. Then, fNIRS systems measure the photonic absorption between two scalps optodes (one emitter and one receiver, forming a channel). This absorption is then used through the modified Beer–Lambert law to calculate the relative changes of oxyhemoglobin and deoxyhemoglobin concentrations for each wavelength. These changes are linked to the local changes in Cerebral Blood Flow (CBF) [67,68]. Thus, fNIRS is similar to fMRI in that it indirectly measures the neuronal activity in a particular area. fNIRS has been shown to be a safe and effective noninvasive brain imaging method, which can be used to study auditory function in patients with hearing loss [69,70,71]. It is little affected by patients’ movements, thus providing an advantage over some other functional imaging techniques [72]. Both cortical sensory areas (e.g., visual, auditory) and integrative areas (e.g., prefrontal cortex) can be analyzed. It also enables a spatially constrained localization of the cortical activity up to a depth of around 15 mm; this represents an advantage over EEG/MEG, where the signals at the scalp result from a complex summation of neuronal activity with an unknown distribution within the brain. A further advantage is that fNIRS can be carried out in a comfortable environment for children, without the noise or intimidating atmosphere created by fMRI scanners. Preparation of the cap and calibration takes about 10 min, which is much shorter than the preparation time usually needed for EEG; this can therefore be less tiresome for children. In addition, the recording time is relatively short, typically lasting about 15 min, unlike the long experimental durations usually required for fMRI and EEG. Finally, although movements can create artifacts in the fNIRS signal, they are less problematic than in EEG and fMRI, thus making fNIRS more suitable for children.

Despite these qualities, fNIRS often raises concerns on whether it is able to measure auditory activity, as the auditory cortex is partly hidden inside the lateral sulcus [73]. Some authors advocate that oxyhemoglobin concentration changes might not be relatable. Indeed, Steinmetzger et al. showed in adult subjects that temporal regions′ oxyhemoglobin responses to speech-like stimuli were less correlated to EEG activity than deoxyhemoglobin, questioning the real interest of the first measure [74]. On the other hand, some authors have already successfully used it for auditory purposes in adults but also in children. In adults, Wiggins et al. [73] showed that fNIRS reliability for auditory speech responses was good to excellent (Pearson’s r = 0.79–0.98) at the group level, even though it was more variable at the individual level. In children, in 2010, Sevy et al. [75] illustrated the capacity of fNIRS to detect auditory speech-induced cortical temporal responses in 100% of their normal-hearing adult population and in about 80% of both their normal hearing and cochlear implanted children population aged from 2 to 19 years. Minagawa-Kawai et al. [76] were able to show, in 4-month-old infants, left-hemispheric lateralization of CBF in response to auditory speech stimuli, whereas non-speech stimuli elicited right-lateralized responses. Two other studies from Telkemeyer et al. using fNIRS suggested that these auditory HDR response lateralizations are quite constant from birth up to 6 months [77,78].

Our protocol uses fNIRS to assess cortical reorganization in UHL. The protocol involves collecting data for a group of children with SSD and a group of age- and gender-matched normally-hearing children. The data from the two groups can then be compared, and analyses can be run to explore the links between fNIRS measures and performance on different tests (e.g., hearing, speech).

### 1.2. Objectives

#### 1.2.1. Primary Objective

The main objective of this protocol is to investigate auditory cortical activation in children with SSD and normally-hearing controls using fNIRS.

#### 1.2.2. Secondary Objectives

The protocol also aims to:

- study the relationship between the oxyhemoglobin/deoxyhemoglobin levels in each auditory cortex during sound stimulation and the scores on different tasks: sound localization, speech-in-noise recognition, language assessment, and quality of life measures.
- describe the relationship between the oxyhemoglobin/deoxyhemoglobin levels in each auditory cortex during sound stimulation and the duration of deafness/age of children with SSD.

### 1.3. Protocol Design

The protocol is for an exploratory, single-center, cross-sectional study, which aims to compare two groups of children: those with SSD and age- and sex-matched normally-hearing controls. There are 30 children in each group, aged 5–16 years.

The children first undergo a baseline evaluation to confirm their inclusion; they then complete two sessions, each lasting maximum 2 h, separated by a gap of up to 3 months.

For the first session (V1), the children undergo auditory and language testing. For the second session (V2), auditory cortical imaging is carried out using fNIRS.

The fNIRS is non-invasive and uses a sensor-bearing cap. The raw intensity values are converted to optical density units and then, using the modified Beer–Lambert Law, to the relative concentrations of oxy- and deoxyhemoglobin. General linear models (GLM) including short channels are used; classical averaging per condition is also feasible. As models leave an inevitable residual variability (error term), which is different across subjects and channels and may present a bias, we prefer to use both approaches: GLM based and simple averaging to compare their results. Averaging is carried out for each condition, and the amplitudes of the peaks and the areas under the curves are calculated to enable comparison between the groups.

Group comparisons are carried out for the binaural performance (speech-in-noise recognition and sound localization ability), language skills, and quality of life (QoL) measures. Further analyses are carried out to determine whether these measures relate to the cortical activity and the extent of functional reorganization.

## 2. Materials and Methods

### 2.1. Eligibility Criteria

#### 2.1.1. Inclusion Criteria

- Children aged 5–16 years
- French speaking
- Covered by French social security
- Able and willing to participate in all sessions with parental written consent.
- Children with SSD have moderate to profound unilateral hearing loss (>40 dB) that has never been treated.
- Control group children have normal hearing (air-conduction thresholds <20 dB).

#### 2.1.2. Exclusion Criteria

- Neurological disorders or other sensory or motor deficits
- Bilingualism
- Medications affecting attention

### 2.2. Measurements

#### 2.2.1. Language Assessment: Perception and Expression

Auditory perception is evaluated using the Categories of Auditory Performance II (CAP II) rating scale from the Nottingham Early Assessment Package [79]. This hierarchical scale assesses auditory perception in everyday environments, and children are given a score ranging from zero to nine.

Speech intelligibility is evaluated using a categorical scale, the Speech Intelligibility Rating (SIR) scale from the Nottingham Early Assessment Package [80]. The clarity and intelligibility of the child’s speech is given a score out of five.

Receptive and expressive language skills are assessed using standardized tests that are appropriate to the age and perceived language level of a child. These tests assess speech comprehension and expression, sentence construction, and executive functions such as attention and working memory.

For children younger than seven, the French test battery EVALO 2–6 short version is used [81]. This involves assessing children through standardized play or activities; the results are either raw scores or standard deviations from the age norm. For children aged seven or above, receptive vocabulary is assessed using the French version of the Peabody Picture Vocabulary Test (PPVT) [82]; this gives a raw score and an age equivalent score. Syntactic and morphosyntactic comprehension are assessed using the E.CO.S.SE test (“échelle de compréhension syntaxico-sémantique”) [83]; this again gives a raw score and age equivalent scores.

Memory span is assessed using the forward and backward conditions of the digit span test from the Wechsler Intelligence Scale for Children-Fourth Edition (WISC-IV) [84]. For this, children are presented with a series of numbers which they are asked to recall in the correct order (forward span) or the reverse order (backward span). This assesses the working memory capacity (forward span) and manipulation skills within working memory (backward span). The digit span is associated with the functional age.

#### 2.2.2. Assessment of Binaural Function

Sound localization is assessed using age-appropriate tasks. For children under the age of seven, a left-right lateralization task with non-linguistic sounds is carried out in a dedicated room [85], and the percentage of errors is recorded. For children aged seven or older, a sound localization test with 12 loudspeakers is used. For this, the child is seated and the loudspeakers are placed in a semi-circular array around the child (1.20 m of distance), each separated by 15°. The child faces the array, with the loudspeakers located from 82.5° on the right to −82.5° on the left [86]. The child hears the sound of a gun shot (presented at 65 dB) and is asked to decide which loudspeaker presented the sound. Responses are made by selecting the corresponding location on a tablet which shows the loudspeaker array. Results are recorded as the degrees of error (15° standard) and the percentage of errors.

Speech-in-noise perception is assessed using age-appropriate tasks, presented in a sound booth. The Common Phrases Test (Common Test version) [87] is used for children up to the age of six, the Hearing in Noise Test (HINT-C) test [88] is used for children aged seven to eleven, and the Marginal Benefit from Acoustic Amplification (MBAA) test [89] is used for children older than eleven. The speech is presented at 65 dB with a fixed SNR of +10 dB and then +5dB. The speech is presented through a loudspeaker placed in front of the child; the noise (environmental noise) is presented though two loudspeakers placed at either side of the child. Results are recorded as the percentage of speech understood.

#### 2.2.3. Quality of Life

QoL is assessed using the KindL^R^ generic QoL scale and the Speech, Spatial, and Qualities of Hearing Scale (SSQ), which is specific to hearing loss [90].

Two scales have age-appropriate versions: the KindL for children aged 3–6 years; the KindL for children aged 7–13 years [91]; the KindL for children aged 14–17 years; the SSQ French version for parents; and the SSQ French version for children.

#### 2.2.4. Auditory Cortical Imaging

**Figure 1:**
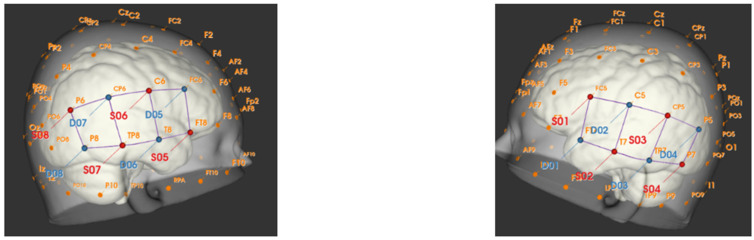
Optode positions and the corresponding cortical landmarks below the scalp; 10 channels (“source-detector” pairs) are available in the left hemisphere and 8 channels in the right hemisphere, the most posterior detector (D08) being used for short channels.

Imaging is performed using fNIRS. This non-invasive functional imaging technique relies on a sensor-bearing cap, which can measure cortical activity during auditory stimulation. We will use the channel distances of 30 mm for all the channels and short channels located at 8 mm from the sources. These short-distance detectors pick up extracerebral signals close to the source and route these signals to an available detector channel through miniaturized fiber optics. Bundles of eight short-distance probes couple to a single detector channel. For this study, optodes are placed with a focus on the auditory cortex and the superior temporal area, as determined using fOLD software (Figure 1). We expect to observe activity mainly in the channels over the Te3 area, the secondary auditory cortex located at the lateral surface of the gyrus, and more posterior temporal areas involved in integrative auditory processing [56,92].

For the recording session, the participant is seated in a sound-proof room where there is no external source of light. An fNIRS cap is chosen according to the subject′s head circumference, and then centered on the head using the left and right preauricular points and the nasion (frontal) and inion (occipital). The optodes and detectors are calibrated using NIRstar software, which visualizes the cap using a model. During calibration, the optode images on the screen are color coded (red/orange/green) according to the signal to noise ratio.

The cortical signals are recorded using NIRstar and begin 1 min before the first sound presentation to obtain the baseline levels of cortical activity. During the recordings, the sounds are presented through noise-cancelling headphones at a level of approximately 60 dB, as measured using a sound level meter. The stimuli include environmental sounds, human voice non-speech sounds, and speech sounds (syllables), which have been validated and used in previous studies [93]. White noise is also presented in separate blocks. The participants are instructed to sit still and listen to the sounds; no specific task is given.

**Figure 2:**
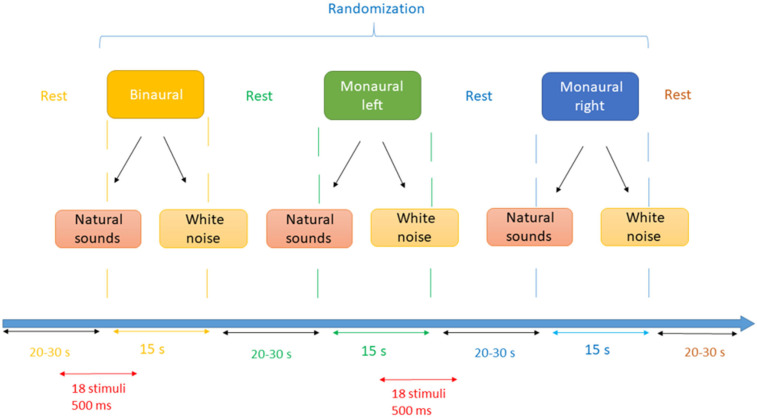
Auditory stimulation blocks and conditions.

The sound stimuli are presented in 15 s blocks, each containing 18 sounds of 500 ms duration, spaced by 300 ms silent gaps. The blocks contain either natural sounds or white noise, and the sounds are presented to either the right ear, the left ear, or binaurally, thus giving a total of six conditions. These are presented in a random order, a total of three times each (Figure 2). The number of blocks could be increased if the results of a pilot study required to increase the SNR. Each 15 s block is preceded by a rest phase of a random duration between 20 and 30 s; this enables the cortical activity to return to baseline levels so that the auditory BOLD response can be determined [94]. Three additional blocks of silence (15 s duration) and two blocks of videos with cartoons are also included in the recording session. The videos provide the children with some entertainment and keep their attention on the screen; they last about 55 s and are preceded and followed by a 15 s rest phase.

At the end of the recordings, which last approximately 20 min in total, the fNIRS cap is removed. The entire session takes up to a maximum of 2 h, including the set-up. The recordings are then analyzed to determine the auditory cortical activity. Indices of inter-hemispheric processing asymmetry are calculated for the different conditions.

### 2.3. Participant Timeline

**Figure 3:**
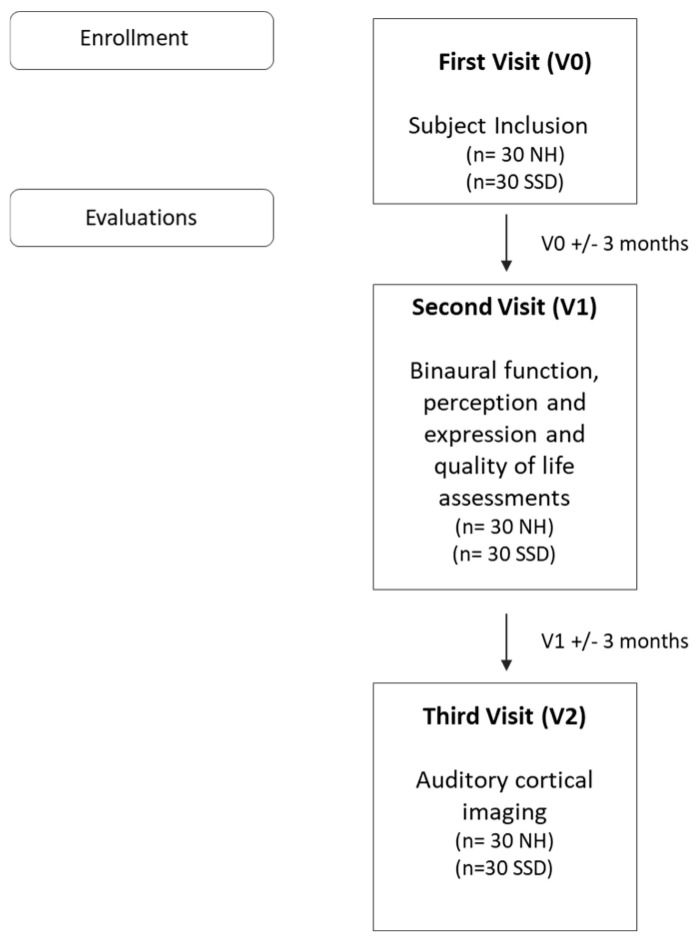
The timeline of procedures during the study.

The timeline of procedures during the study is shown in Figure 3.

#### 2.3.1. Number of Subjects

Thirty children with SSD and thirty normally-hearing children.

#### 2.3.2. Recruitment

Children with SSD are consecutively recruited by physicians at the ENT department, Toulouse Hospital, during follow-up appointments or when diagnosing hearing loss. There are currently estimated to be more than 110 patients who are being followed up for SSD at the hospital, of whom at least 50 are not undergoing rehabilitation and fulfill the study’s eligibility criteria, thus ensuring the feasibility of the study. Normally-hearing children are recruited using posters displayed in the ENT department and pediatric hospital waiting rooms. Before including a potentially eligible child with SSD, a control is sought who is of the same sex and as close in age as possible. An age difference of more than a year is not accepted. Children with SSD are not included if no suitable control can be found. The recruitment continues until 30 children with SSD have been matched to 30 controls.

#### 2.3.3. Data Collection and Management

For each participant, information is collected using a case report form (CRF) that is completed by the investigators. The CRF includes the child’s identity, clinical observations, and medical follow-up data of relevance to the study. This document is kept in the ENT department, Toulouse Hospital. The study data is kept on a computer, with data entry carried out by the investigators and their colleagues. The patients′ CRFs are reviewed on-site prior to data entry, which is then carried out according to procedures determined by the study methodologist, statistician, and principal investigator. The imaging data is saved on an external storage device (CD, DVD) in compliance with the study′s anonymity requirements. This device is attached to the CRF for archiving. The data are kept for a minimum of 15 years.

### 2.4. Statistical Analysis

As this is an exploratory study, descriptive statistics are run for the groups of children to summarize the primary outcome measures: the changes in oxyhemoglobin, deoxyhemoglobin, and their ratio.

The associations between these fNIRS measures and scores on the auditory, speech, and quality of life assessments are also analyzed for each group.

The Matlab-based Homer2 program is used to preprocess the fNIRS signals to obtain curves that can be further analyzed. The preprocessing involves motion correction, band-pass filtering, and principal component analysis (PCA), which detects the greatest amount of spatial covariance across all spatially distributed channels and then removes those components from all channels [95]. The scalp-coupling index, which is used as a measure of the prominence of the photoplethysmographic cardiac signal [96], will be used to detect bad channels.

**Figure 4:**
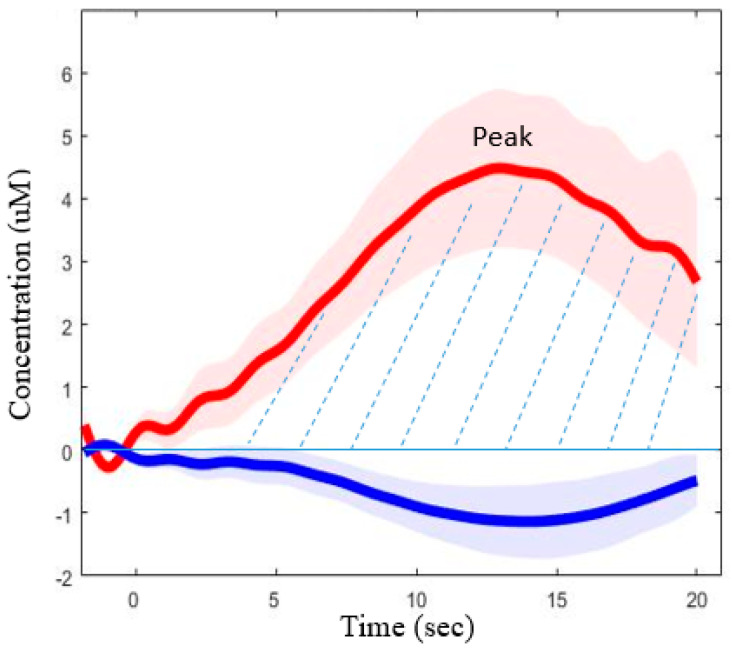
A typical fNIRS curve. The peak amplitude and the area under the curve (dotted lines) are extracted for further statistical analysis.

The preprocessing pipeline is: raw measurement → transformation to optical density → bad channel rejection→ transformation to concentration → PCA → motion correction → band-pass filter. At the first stage, the fMRI-like general linear model analysis will be applied [97] to test the differences between the conditions. Functional responses are calculated as block average waveforms for each condition. Various parameters will then be extracted from the waveforms, using Matlab scripts, for further statistical analysis. The two main parameters are the peak value and the area under the curve (Figure 4). We will use Bonferroni correction for the family-wise error rate (FWER) and the Benjamini–Hochberg correction for the false-discovery rate (FDR).

In a second step, the relative activity levels in the two hemispheres are calculated.

The asymmetry index (AI) is calculated for each monaural condition using the formula: AI = (Contralateral hemisphere activity − Ipsilateral hemisphere activity)/(Contralateral hemisphere activity + Ipsilateral hemisphere activity). AI values are positive when there is greater contralateral activity, and negative when there is greater ipsilateral activity.

**Figure 5:**
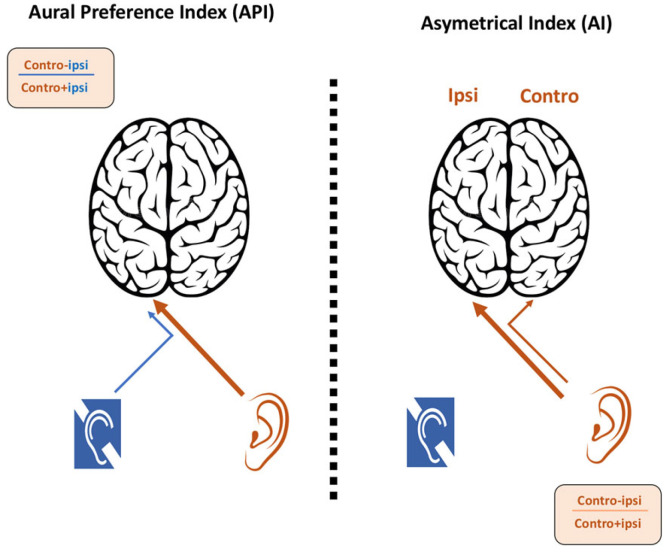
Illustration of the asymmetry index and aural preference index.

The aural preference index (API) is calculated for each hemisphere using the formula: API = (Contralateral ear condition − Ipsilateral ear condition)/(Contralateral ear condition + Ipsilateral ear condition). API values are positive when the contralateral ear has a greater influence (Figure 5).

The within- and between-group analyses are carried out using STATA and R software. Between-group comparisons are run using generalized mixed-effect models, after the data have been checked, outliers corrected, and the data ‘frozen’.

Descriptive statistics are run to summarize the characteristics of the two study groups. They are also run to describe the primary outcome measures: the distribution of oxyhemoglobin and deoxyhemoglobin levels in the auditory cortices during sound stimulation (corresponding to the primary objective). These analyses are carried out for each participant group, and the measures of central tendency (mean, median) and dispersion (range, standard deviation, inter-quartile range) are recorded. Tests (Shapiro–Wilk) are also run to assess the normality of the distribution.

Further analyses are run to assess the association between the fNIRS measures (oxyhemoglobin/deoxyhemoglobin levels and interhemispheric indices) and scores on the auditory, language, and quality of life assessments (secondary objective). These are run for each group of children using Mann–Whitney tests or Spearman′s rank correlation tests, according to the categorical or continuous nature of the variable.

Spearman’s rank correlation tests are also run to investigate the relationship between the fNIRS measures (oxyhemoglobin/deoxyhemoglobin levels and interhemispheric indices) and the ages of the children and duration of deafness in the SSD group (secondary objective).

### 2.5. Ethics and Dissemination

All of the authorizations required by French legislation were obtained. The study was approved by the Ethics Committee in May 2018.

#### 2.5.1. Trial Registration

NCT04043910 in Clinical Trials.gov

#### 2.5.2. Protocol Version

Version 2, 15 July 2020.
Protocol amendments
The protocol was amended (change the phone number of the contact person in the recruitment poster) and approved by the Ethics Committee in March 2021.

#### 2.5.3. Confidentiality

In accordance with the regulations, those with direct access to the data take all necessary precautions to ensure the confidentiality of information relating to the study participants, in particular their identity and results.

During the research or at its conclusion, the data collected is made anonymous. For the children with SSD, only the first letter of their name and surname are recorded, along with a coded number indicating the order of their inclusion, and the letter S (for SSD). For the matched control children, the same code is assigned plus the letters NH. For each child, the investigators ensure that the child’s legal guardians provide written consent for the team to access the child’s data that is necessary for the research. No information enabling the identification of participants is communicated to third parties other than those legally authorized to hold this information (and who are bound by professional secrecy).

## 3. Working Hypothesis

The experimental hypothesis is that both the AI and API indices are altered in children with SSD. It is hypothesized that the API index is negative for the hemisphere contralateral to the deaf ear, corresponding to an abnormal ipsilateral aural preference. This would be in line with previous studies that have described an aural preference syndrome in adults with SSD [44,56,57]. The API values are expected to relate to the duration of deafness and/or the age of the children.

For the AI, it is hypothesized that stimulation of the better ear shifts the AI toward the ipsilateral auditory cortex, as found in a study on adults with SSD [56]. This shift is thought to disrupt the neuronal interactions within the ispilateral auditory cortex. We hypothesize that the lateralization of auditory processing, expressed by the AI, mainly relates to the contralateral sound field rather than the contralateral ear per se [41,42,43]. In consequence, we expect the children’s AI values to correlate positively with spatial hearing performance (i.e., sound localization scores).
